# Supplementary material for: A Comparative Transcriptomic Meta-Analysis Revealed Conserved Key Genes and Regulatory Networks Involved in Drought Tolerance in Cereal Crops
Source: Int J Mol Sci. 2021 Dec 2;22(23):13062. doi: 10.3390/ijms222313062 (PMC8657901; doi:10.3390/ijms222313062)
Supplement: Supplementary file 1 [file ijms-22-13062-s001.zip › Legends of Supplementary Materials.pdf]

Supplementary Materials

Figure S1. Scatter plots of mean log2(counts-per-million) *vs* log2(fold change) for the differential expression analyses of treated *vs* control conditions for the eight experiments (four species, two genotypes each). Transcriptional changes due to drought treatment are presented for susceptible and tolerant cultivars in the four species. Genes with an FDR smaller than 0.05 are represented in red for sensitive genotypes and in blue for tolerant genotypes.

Figure S2. Pie charts of the distribution per class of the DEG-TFs identified in the *SGR* / photosynthesis anti-correlated clusters in the four species. Classes are as those defined in Plant TFDB [86]. Bd: *B. distachyon;* Hv: *H. vulgare;* Os: *O. sativa*; Zm: *Z. mays*.

Table S1. RNA libraries sequencing, filtering and mapping statistics. For each RNA library, the number of raw reads, the number and percentage of reads after filtering out the adapters and low-quality sequences, and the number and percentage of reads mapped to respective reference genome are reported.

Table S2. DEGs orthology analyses statistics. Number and percentage of DEGs with orthology found in rice predicted genes from the four species were reported.

Table S3. The 142 DEGs that resulted common to the 4 species, following the orthology with the rice genome. For each gene, the log2(Fold Change) for sensitive and tolerant genotypes and the corresponding class are shown. The LFC of the genes reported in Todaka et al. (2017) under Sds treatment is shown. The 69 genes putatively involved in tolerance are marked.

Table S4. Differential expression analyses result for the genes with an orthologous in rice predicted genes. Rice genes with orthology to genes found as DE at least in one of the four datasets were listed in the first column. For each of the rice genes, the DE analyses results of the orthologous gene in the eight genotypes were reported only if FDR is smaller than 0.05. For each of the four species we reported the ortholog to rice gene, the log2(Fold Change) for sensitive and tolerant accessions (only if FDR<0.05) and the class according to classification specified in Materials and Methods paragraph.

Table S5. Differential expression analyses results and description of the 69 genes whose ortholog were found as differently regulated in sensitive and tolerant accession in all the analyzed species. For each gene we reported the ortholog to rice gene, the log2(Fold Change) for sensitive and tolerant accessions (only if FDR<0.05) and the class according to classification specified in Materials and Methods paragraph. Moreover, the position on rice reference genome and the description of each rice ortholog gene were reported. Among the 69 rice genes, the Senescence Associated Genes (SAGs), following the data from Lee et al. 2017, are marked.

Table S6. List of the rice 69 CDT and TF encoding genes used for the gene co-expression network. These TF genes resulted differentially expressed in the experiment used for validation [44]. The corresponding gene expression RPKM values from [44] are reported.

Table S7. Network analysis statistics of the gene co-expression network created using Pearson's correlation value $|r| \geq 0.9$ (787 genes). The r correlation value of each gene with *Psao* (OS_28) and *SGR* (Os_58) are reported.

Table S8. List of the 43 CDT genes present in the gene co-expression network related to the osmotic stress experiment. Pearson's correlation (r) values of each gene in respect to *PsaO* and *SGR* are shown. The 26 and 16 CDT genes positively correlated with either *PsaO* or *SGR* are highlighted in green and purple, respectively.

Table S9. List of the 3000 upstream sequences of the rice CDT genes, used for the *cis*-element analysis.

Supplementary File S1. Differential expression analyses result of treated *vs* control conditions for the eight experiments (four species, two genotypes each). Each DE analysis is reported in one spreadsheet.

Supplementary File S2. DEGs classification and orthology to rice genes. For each DEG in the four datasets the log2(Fold Change) for sensitive and tolerant accessions, the class according to classification specified in Materials and Methods paragraph, and the putative rice ortholog were reported.

Supplementary File S3. Annotation of DEG transcription factors in the four datasets. TF families were assigned as indicated in Materials and Methods. Rice ortholog IDs, logFC (log2 fold-changes) and DEG classes are also included.

Supplementary File S4. The 69 selected genes and DEG transcription factor genes used for k-means cluster analyses. The assigned gene name, the cluster they belong and the cluster score, obtained by correlating each gene with the cluster core, are reported. The genes belonging to the anticorrelated clusters related to *STAY GREEN* and photosynthesis related genes are highlighted in red and green, respectively.

Supplementary File S5. Transcription factor encoding genes belonging to the anticorrelated clusters related to *STAY GREEN* (red) and photosynthesis related genes (green) in the four species. For rice genes, the presence in the CGN and r values of the correlation with *PsaO* and *SGR* in the GCN are reported.

Supplementary File S6. Correlation matrix of 797 genes (69 CDT genes and TF DEGs listed in Table S6), related to validation experiment (Baldoni et al. 2016). Pearson correlation values (r) with $p < 0.05$ are reported.

Supplementary File S7. Analysis of the presence of *cis*-regulatory binding sites for the identified TF classes in the 3000 bp genomic regions upstream of the rice CDT genes.
