# Supplementary figures and images for: A Comparative Transcriptomic Meta-Analysis Revealed Conserved Key Genes and Regulatory Networks Involved in Drought Tolerance in Cereal Crops
Source: Int J Mol Sci. 2021 Dec 2;22(23):13062. doi: 10.3390/ijms222313062 (PMC8657901; doi:10.3390/ijms222313062)

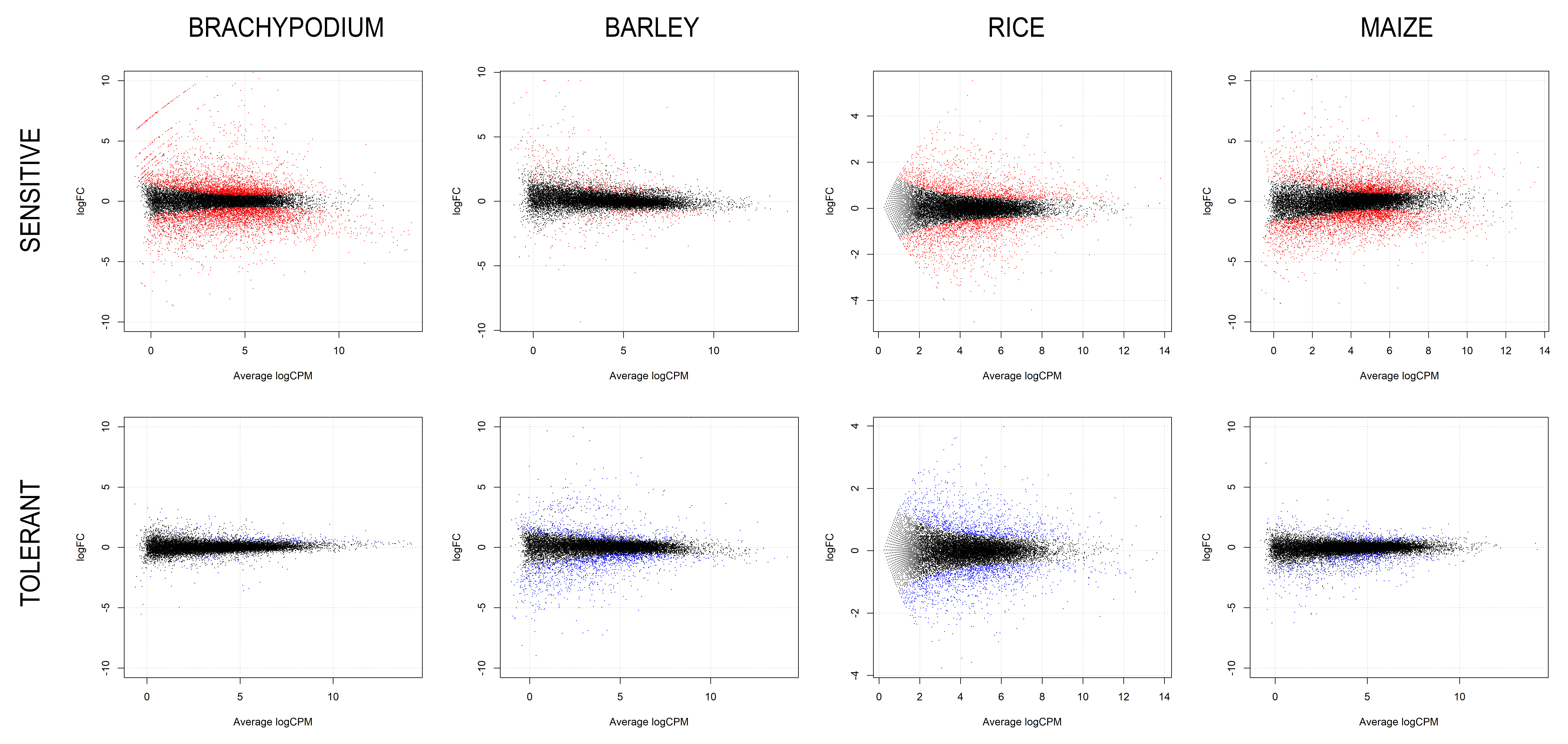

Supplement: Supplementary file 1 [file ijms-22-13062-s001.zip › Figure S1.tif]

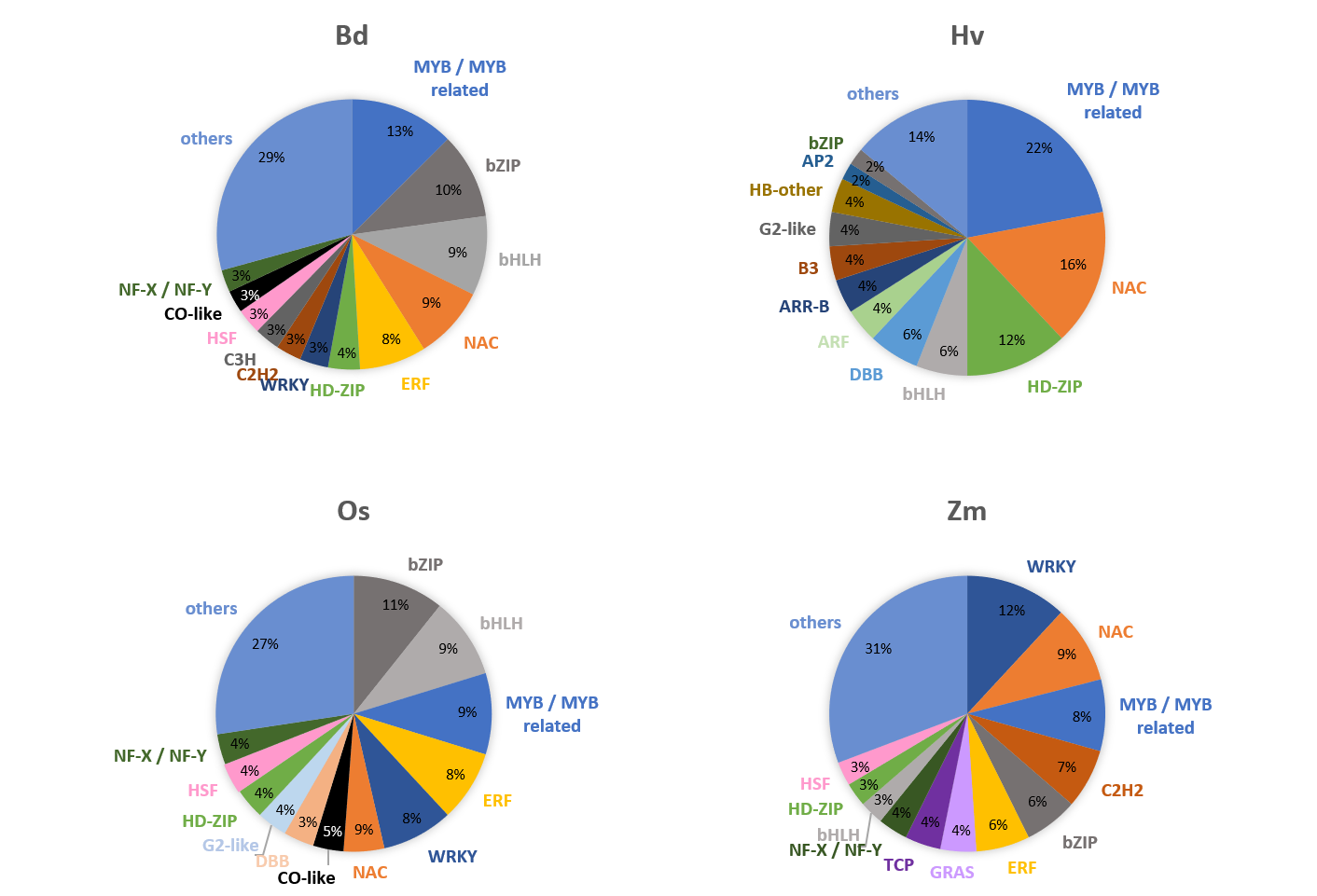

Supplement: Supplementary file 1 [file ijms-22-13062-s001.zip › Figure S2.png]
